# *In vitro* Examination of Piezo1-TRPV4 Dynamics: Implications for Placental Endothelial Function in Normal and Preeclamptic Pregnancies

**DOI:** 10.1152/ajpcell.00794.2024

**Published:** 2024-12-09

**Authors:** Hanna H Allerkamp, Alexander I Bondarenko, Ines Tawfik, Nilüfer Kamali-Simsek, Monika Horvat Mercnik, Corina T Madreiter-Sokolowski, Christian Wadsack

**Affiliations:** 1 Department of Obstetrics and Gynecology, Medical University of Graz, Graz, Austria; 2 Molecular Biology and Biochemistry, Medical University of Graz, Graz, Austria; 3 BioTechMed-Graz, Austria

**Keywords:** placenta, ion channels, endothelium, preeclampsia, nitric oxide

## Abstract

Mechanosensation is essential for endothelial cell (EC) function, which is compromised in early-onset preeclampsia (EPE), impacting offspring health. The ion channels Piezo-type mechanosensitive ion channel component 1 (Piezo1) and Transient receptor potential cation channel subfamily V member 4 (TRPV4) are co-regulated mechanosensors in ECs. Current evidence suggests that both channels could mediate aberrant placental endothelial function in EPE. Using isolated feto-placental ECs (fpECs) from early control (EC) and EPE pregnancies, we show functional co-expression of both channels and that Ca^2+^ influx and membrane depolarization in response to chemical channel activation is reduced in EPE fpECs. Downstream of channel activation, Piezo1 alone can induce phosphorylation of endothelial nitric oxide synthase (eNOS) in fpECs, while combined activation of Piezo1 and TRPV4 only affects eNOS phosphorylation in EPE fpECs. Additionally, combined activation reduces the barrier integrity of fpECs, also with a stronger effect on EPE fpECs. This implies altered Piezo1-TRPV4 co-regulation in EPE. Mechanistically, we suggest this to be driven by changes in the arachidonic acid metabolism in EPE fpECs as identified by RNA-Seq. Targeting of Piezo1 and TRPV4 might hold potential for EPE treatment options in the future.

## Graphical abstract

**Figure:**
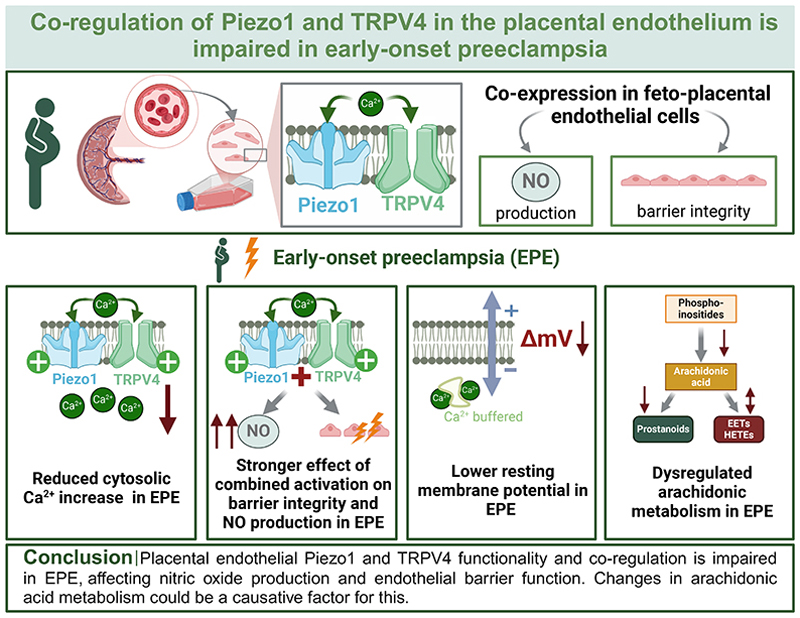

## Introduction

Throughout pregnancy, the placenta plays a critical role in facilitating the connection and optimal gas and nutrient exchange between mother and fetus under minimal resistance within the vascular system. Thereby, the successful development and proper functioning of the placenta have a major influence on the outcome of the pregnancy. From the end of the first trimester, the villi of the human placenta become immersed in maternal blood, which fills the intervillous space. At the same time, the feto-placental blood vessels, which are responsible for transporting blood to and from the umbilical cord, develop in close proximity within these villi (1).

Preeclampsia (PE), a condition affecting 2-10% of pregnancies worldwide, is a significant contributor to maternal and perinatal morbidity and mortality (2, 3). Maternally, PE manifests as the onset of hypertension and proteinuria or the onset of PE-related symptoms without proteinuria >20 weeks of gestation (4). Early-onset PE (EPE) in particular (maternal symptoms apparent <34 weeks of gestation) is associated with severe neonatal morbidity and often accompanied by reduced fetal growth (5). The etiology of PE is multifactorial, and current treatment options remain limited, often resulting in early delivery as the only “cure”. Generally, the development of EPE is believed to occur in two main stages, resulting from failed placentation and ultimately leading to placental damage and oxidative stress, the release of pro-inflammatory factors, and systemic endothelial activation (6, 7).

While PE research over the last decades has focused on maternal endothelial dysfunction, our knowledge of the effects on the placental vasculature remains limited. With respect to endothelial barrier integrity, endothelial cells from PE placentae show ultrastructural signs of barrier disruption (8). In addition, several *ex vivo* studies have demonstrated that chorionic arteries from PE placentae show altered vascular reactivity with an increased vasomotor tone (9). These changes may ultimately lead to higher resistance within the placental vascular bed (10). This is also reflected *in vivo* by changes in the umbilical Doppler waveform and reduced placental vascular indices (11–13). An increase in placental resistance results in an elevated afterload for the developing fetal heart (14). Moreover, PE has been identified as an independent risk factor that increases the risk of the offspring developing cardiovascular morbidities in later life by up to 40% (15–17). This indicates that improving placental vascular function in PE would be beneficial for the long-term cardiovascular outcome of the offspring.

Ion channels are crucially involved in the regulation of vascular tone and integrity (18). Endothelial dysfunction in a wide spectrum of pathologies is commonly associated with and preceded by the deregulation of Ca^2+^-dependent and Ca^2+^-permeable endothelial ion channel function (19–21). Impaired Kv7 channel functionality in smooth muscle cells has been exemplified in placental vessels affected by PE (22), but knowledge of deregulation of endothelial ion channels in PE is limited. Functionally, placental vessels in PE demonstrate reduced flow-mediated endothelial-dependent vasodilation (23), suggesting impaired shear stress sensing mechanisms or signaling. The mechanosensitive ion channel Piezo-type mechanosensitive ion channel component 1 (Piezo1) has recently been shown to act as a shear stress sensor in feto-placental ECs (fpECs), with potential implications for pregnancy disorders (24–26). In other organs, a reciprocal relationship between Piezo1 and the Transient receptor potential cation channel subfamily V member 4 (TRPV4) has been demonstrated (27–31). Interactions between the two channels appear to affect the pattern of cytosolic calcium changes, which acts as an important second messenger (28, 29, 31).

Importantly, both channels have been shown to be involved in the vasodilation of placental vessels, at least in part via the major vasodilator nitric oxide (NO) (25, 32). In placentae affected by PE, both NO production and bioavailability have been shown to be impaired at multiple levels (33–35). This impairment is thought to contribute to the more vasoconstrictive phenotype observed in PE. With regard to endothelial barrier disruption as another hallmark of PE, both channels have been shown to mediate the organization of adherens junctions and F-actin in human umbilical vein endothelial cells (HUVECs) (28). Interestingly, TRPV4 itself is mechanosensitive, yet it can also be activated by various pro-inflammatory bioactive lipids, including downstream metabolites of arachidonic acid (AA) (36). In PE, placental AA metabolism is altered (37), and relevant downstream metabolites are elevated in placental tissue (38).

Taken together, the current evidence points to both channels as interesting candidates for mediating aberrant placental endothelial function in EPE. More specifically, we hypothesize that the functionality of Piezo1 and TRPV4 is impaired in EPE and that, mechanistically, the activation of TRPV4 by altered AA metabolites in EPE may affect Piezo1 functionality. This study aims to investigate the functionality and interaction of Piezo1 and TRPV4 in early control (EC) and EPE fpECs in order to better understand the links between EPE and dysfunction of the placental endothelium.

## Materials and Methods

### Sample collection

Placentae were obtained immediately after delivery of singleton pregnancies at the Department of Obstetrics and Gynecology, Medical University of Graz, Austria. Collection and sample processing were approved by the ethics committee of the Medical University of Graz, Austria (29-319 ex 16/17) and voluntary detailed consent was taken from all study participants. Exclusion criteria were maternal systemic disease, other pregnancy complications, nicotine use during pregnancy, and a body mass index of <18 or >27 before pregnancy. The control samples were divided into two control groups: those delivered at ≥38 weeks of gestation called term controls (TC) and those delivered at <38 weeks of gestation called early controls (EC), the latter being age-matched to the early-onset PE group (EPE). EPE was diagnosed by a qualified clinician according to ACOG guidelines (new–onset hypertension with a blood pressure of ≥140/90 mmHg plus organ manifestation with onset of maternal symptoms <34 weeks of gestation (39)). Detailed characteristics of the study participants are shown in Table 1.

### Isolation and cultivation of fpECs

Primary fpECs were obtained and cultured from chorionic arteries of human term placentae according to an established protocol (40). First, arteries were dissected from the surface of the chorionic plate, cannulated, and rinsed with Hank’s balanced salt solution (HBSS; Gibco, ThermoFisher Scientific, Waltham, USA). FpECs were then extracted by perfusion of the selected arteries with 20 ml of pre-warmed HBSS containing 0.1 U/ml collagenase, 0.8 U/ml dispase II (Roche, Basel, Switzerland), and 10 mg/ml penicillin/streptomycin (Gibco) at 2.5 ml/min. Cells were collected in 10 ml fetal calf serum (FCS; Gibco), centrifuged at room temperature (900 rpm, 7 min), and resuspended in endothelial cell growth medium (C-22220, Promocell, Heidelberg, Germany) supplemented with endothelial cell growth factor, hydrocortisone, epidermal growth factor (C-39220, Promocell), 0.05 mg/ml gentamicin (Gibco) and 10% defined FCS (Gibco). The cells were then plated on 12-well plates coated with 1% gelatin (Sigma Aldrich, St. Louis, USA) and incubated at 37°C in 12% O_2_ and 5% CO_2_. Upon reaching sufficient confluence, the cells were transferred to cell culture flasks, maintained under the same conditions, frozen, and stored in liquid nitrogen until further use. The identity and purity of the fpECs were confirmed by flow cytometry. Therefore, fpECs were harvested from flasks using TrypLE (ThermoFisher Scientific). A minimum of 5x10^5^ viable cells/tube were resuspended in 3% FCS – HBSS solution for 10 min at room temperature to block Fc-receptors and reduce non-specific binding, then fixed and permeabilized using the BD Cytofix/Cytoperm kit (BD Biosciences, New Jersey, USA). Staining was performed according to the manufacturer’s instructions. A minimum of 3x10^4^ events were counted per sample. The endothelial markers CD31 (Biolegend, San Diego, USA; Cat #303118; RRID:AB_2247932) and VE-cadherin (BD Biosciences; Cat #743705; RRID:AB_2741685), and the mesenchymal marker vimentin (Biolegend; Cat #699305; RRID:AB_2888889) were used for quality control. CD90 (Biolegend; Cat #328132; RRID:AB_2566341), Col1A1 (NovusBio, Centennial, USA; Cat #NB600-408AF700; RRID:AB_3194932), smooth muscle actin (ThermoFisher Scientific; Cat #50-9760-82; RRID:AB_2574362) and transgelin (NovusBio; Cat #NBP2-47689PE; RRID:AB_3313812) were used to further assess potential fibroblast or muscle cell contamination. Isotype controls corresponding to each fluorochrome used in the experiment were used to detect non-specific positive signals. Cells were separated by size using forward and side scatter (FSC and SSC, respectively), followed by doublet discrimination. In the final step, cells for each marker were plotted as histograms to detect positive signals. Measurements were performed using a CytoFLEX flow cytometer (Beckman Coulter, Brea, USA) and analyzed using FlowJo™ v10.10 software (RRID:SCR_008520) for gate setting and data analysis. The fpEC isolations used in this study showed the following mean marker expression confirming endothelial identity and purity: CD31 95.41% (SD 10.24%), VE-cadherin 97.09% (SD 4.38%), vimentin 99.29% (SD 0.98%), CD90 17.87% (SD 9.18%), Col1A1 0.046% (SD 0.065%), smooth muscle actin 0.30% (SD 0.89%) and transgelin 8.29% (SD 11.70%).

### *In-situ* hybridization (ISH)

The RNAscope™ Multiplex Fluorescent V2 Assay (Advanced Cell Diagnostics, Newark, USA) was used according to the manufacturer’s instructions. FpECs (n = 3 biological replicates/group) were seeded onto 1% gelatin-coated glass chamber slides and grown to 80-90% confluence. Cells were fixed in 4% paraformaldehyde for 30 min at room temperature, dehydrated through an ascending ethanol series and stored in 100% ethanol at -20°C until use. On the day of the experiment, briefly, cells were rehydrated at room temperature, peroxidase was blocked with hydrogen peroxide for 10 min, and cells were treated with Protease III diluted 1:15 in phosphate buffered saline (PBS). For tissue sections (n = 3 biological replicates/group), tissue samples containing the entire cross-section of the placenta were taken, washed in PBS, fixed in 4% paraformaldehyde at 4°C overnight, and embedded in paraffin. 5 µm thick tissue slices were air-dried on SuperFrost® Plus glass slides (ThermoFisher Scientific) overnight and stored at room temperature until use. On the day of the experiment, briefly, slides were baked at 60°C for 1 hour and deparaffinized. Peroxidase was blocked with hydrogen peroxide for 10 min at room temperature and slides were placed in pre-heated Target Retrieval in a Braun FS 3000 steamer (Braun, Kronberg im Taunus, Germany) at 95-99°C for 15 min. Slides were then dried and treated with Protease Plus at 40°C for 20 min in a RapidFISH Slide Hybridizer oven (Boekel Scientific, Feasterville, USA). The following probes were used: Hs-PIEZO1 (#485101), Hs-TRPV4-C3 (#452221-C3), Hs-PECAM1-O1-C2 (#487381-C2), and the 3-plex negative and positive control probes (#320871; #320861). Probe incubation and amplification steps were performed according to the manufacturer’s guidelines. Probes were detected with TSA Vivid dyes (Tocris, Bio-Techne Ltd., Abingdon, UK) 520 (diluted 1:1500), 570 (diluted 1:750), and 650 (diluted 1:750). Nuclei were stained with DAPI, slides were mounted using fluorescence mounting media and stored at 4°C until imaging.

Imaging of tissue sections was performed using an AxioScan 7 slide scanner (Zeiss, Oberkochen, Germany) with a 20x objective, and 4 areas (4000x4000 pixels) of each scan were used for analysis. Imaging of isolated fpECs was done with an Olympus SLIDEVIEW VS200 slide scanner (Olympus, Tokyo, Japan) with a 20x objective. Analysis was conducted with ImageJ (version 1.53a, NIH; RRID:SCR_003070). To investigate mRNA expression within endothelial cells only, *PECAM1* was used as an EC marker on tissue sections. The *PECAM1* channel was thresholded and the analyze particles function was used to measure the *PECAM1^+^* area and create regions of interest. Next, the *PIEZO1* and *TRPV4* channels for each image were thresholded and the analyze particles function was used in a custom written macro to count the number of positive particles within the *PECAM1^+^* area. To quantify the co-localization of *PIEZO1* and *TRPV4* within the *PECAM1^+^* area the EzColocalization plug-in (41) was used to measure the threshold overlap score (TOS) after clearing the image around the *PECAM1^+^* area for tissue sections. For isolated fpECs, those measurements were performed on the total area and the number of nuclei was counted as a reference in each image using the analyze particle function on the DAPI channel.

### Quantitative real-time PCR (qPCR)

FpECs (n = 4-5 biological replicates/group) were harvested by scraping and stored in QIAzol Lysis Reagent (Qiagen, Venlo, Netherlands). RNA was isolated using the miRNeasy Mini Kit (Qiagen) and cDNA was synthesized with the LunaScript® RT SuperMix Kit (New England Biolabs, Ipswich, UK) according to the manufacturers’ guidelines. qPCR was conducted with the CFX384 Touch Real-Time PCR Detection System (Bio-Rad, Hercules, USA) using the Luna Universal qPCR Master Mix (New England Biolabs) with the following QuantiTect primer assays (Qiagen): *HPRT1* (#QT00059066) and *RPL30* (#QT00056651) used as housekeeping genes, *PIEZO1* (#QT00088403), and *TRPV4* (#QT00077217). DNA amplification was performed for 40 cycles with an initial pre-incubation step of 15 min at 95°C, followed by 15 s at 94°C, 30 s at 55°C and 30 s at 72°C. No amplification and no template controls were included in each run and melting curve analysis was conducted to ensure specificity. Measurements were performed in triplicates and averaged for each sample. Threshold cycle (CT) values were corrected for primer efficiency and samples were compared using the 2^(-ΔΔCT)^ method. Statistics were performed on ΔCT values.

### Bulk RNA-Seq and analysis

1x10^6^ fpECs per sample (n = 5-7 biological replicates/group) were harvested using TrypLE and stored in QIAzol Lysis Reagent. RNA was isolated using the miRNeasy Micro Kit (Qiagen) and quality was assessed using a 2100 Bioanalyzer (Agilent, Santa Clara, USA). A NEBNext Poly(A) mRNA Magnetic Isolation Module (New England Biolabs) was used to extract mRNA from total RNA followed by library preparation with a NEBNext Ultra II Directional RNA Library Prep Kit for Illumina (New England Biolabs). The library was sequenced on a NextSeq2000 P2 PE100 (Illumina, San Diego, USA), generating >20 million 100 bp paired-end reads per sample. Raw reads were processed on the Galaxy server of the Medical University of Graz (Galaxy Version 0.73). The FastQC tool (42) was used for quality check and the Trimmomatic tool (43) was used to generate clean reads by removing the adapter-containing and low-quality reads. The clean reads were mapped using the RNA STAR tool (44) against the built-in human reference genome GRCh38 and the aligned reads were counted using the htseq-count tool (45) with the human GRCh38 data set retrieved from Ensemble (https://ftp.ensembl.org/pub/release-90/gtf/homo_sapiens/, accessed 08 Nov 2023). The DESeq2 tool (46) was used on a publicly available Galaxy server (Galaxy Version 2.11.40.8) to generate normalized counts and identify overall differentially expressed genes between EPE and EC fpECs. Further analysis and visualization was conducted using the RStudio Server (2021.09.2 Build 382). For the comparison of expression levels of gene sets of interest, genes with normalized counts >0 in at least 90% of the samples were included in the analysis. ROAST gene set tests (47) were used to compare the EPE and EC group using the fry() function of the edgeR package (version 3.40.0; RRID:SCR_012802). This function provides a mixed p-value and an up/down p-value. The mixed p-value provides information whether the majority of the genes in the set of interest are generally differentially expressed in any direction. The up/down p-value provides information whether the majority of genes in the set are differentially expressed in the specified direction. These p-values are specified in the respective results section. Heat maps were generated using the pheatmap package (version 1.0.12; RRID:SCR_016418).

### Ca^2+^ imaging experiments

For live-cell imaging experiments, 3-4x10^5^ fpECs (n = 3 biological replicates/group) were seeded on 30 mm glass coverslips coated with 1% gelatin 2 days before the experimental day and grown under the same conditions as described above. Immediately prior to experiments, fpECs were kept in EH-loading buffer (135 mM NaCl, 5 mM KCl, 2 mM CaCl2, 1 mM MgCl2, 10 mM HEPES, 2.6 mM NaHCO3, 440 μM KH2PO4, 340 μM Na_2_HPO_4_, 10 mM D-glucose, 0.1% vitamins, 0.2% essential amino acids, and 1% penicillin-streptomycin, pH adjusted to 7.4) at room temperature. During the measurements, the cells were continuously perfused with a Ca^2+^-containing buffer consisting of 145 mM NaCl, 5 mM KCl, 2 mM CaCl2, 1 mM MgCl2, 10 mM D-glucose, and 10 mM HEPES, pH adjusted to 7.4. To measure cytosolic Ca^2+^ levels, cells were incubated with the fluorescent cytosolic Ca^2+^ indicator acetoxy-methyl-ester (Fura-2AM) (TEFLabs, Austin, USA) for 30 min in EH-loading buffer, as described previously (48). After incubation with Fura-2AM, fpECs were washed twice with EH-loading buffer. An NGFI AnglerFish C-Y7G imager equipped with a Nikon CFI Super Fluor 40x oil NA 1.3 objective and a NGFI PS9D perfusion system (NGFI, Graz, Austria) was used. Fura-2AM was illuminated with 340 nm and 380 nm, and emission was captured at 515 nm (495dcxru; Omega Optical, Brattleboro, USA). After the acquisition of baseline measurements for 2 min either the Piezo1 agonist Yoda1 (10 μM; Sigma-Aldrich, Burlington, MA, USA), the TRPV4 agonist GSK1016790A (GSK; 50 nM; Calbiochem, San Diego, USA) or 100 μM ATP (Roth, Karsruhe, Germany) as a positive control was added to the perfusates as indicated in the results section. Concentrations of the agonists were informed by ranges reported in the literature (24, 28) and tested in preliminary experiments. The measurements were recorded using live-acquisition software, background subtracted using a background region of interest and corrected for bleaching using an exponential decay fit. Results are shown as the ratio of F340/380.

### Electrophysiological recordings

Membrane potential was recorded (n = 3-4 biological replicates/group and setup) by using the gramicidin-perforated patch-clamp technique in a current-clamp mode essentially as described previously (49, 50). Borosilicate glass pipettes were pulled with a Narishige puller (Narishige Co. Ltd, Tokyo, Japan), fire-polished, and had a resistance of 4–6 MΩ when filled with a pipette solution containing (in mM): 140 KCl, 0.2 EGTA, 1 MgCl_2_, 10 HEPES (pH adjusted to 7.2 using KOH). For membrane perforation, gramicidin (100 μg/ml) was added to the patch pipette immediately before use. The pipette tip was filled with a solution containing (in mM): 100 K-Gluconate, 40 KCl, 0.2 EGTA, 10 HEPES. The bath solution contained (in mM): 140 NaCl, 5 KCl, 1.2 MgCl_2_, 10 HEPES, 8 glucose, 2.4 CaCl_2_. The experiments were conducted at room temperature. For intracellular Ca^2+^-chelation, fpECs were pre-incubated in a bath solution containing BAPTA-AM (20 µM) at 37°C for 20 min (50). Yoda1 (5 µM) and/or GSK (10 nM) were administered to the fpECs by bath perfusion as indicated in the results section. Membrane potential was recorded using an Axopatch 200B amplifier (Axon Instruments, Foster City, USA). The signals obtained were digitized with a sample rate of 10 Hz using a Digidata 1322A A/D converter (Axon Instruments). Data acquisition and analysis were performed using Clampex (version 8.2) and Clampfit (version 11.2; software from pClamp, Molecular Devices, San Jose, USA; RRID:SCR_011323).

### Western Blot (WB)

WB was used to quantify the level of endothelial NO synthase (eNOS) and phosphorylated eNOS at Ser1177 (p-eNOS) as a measure of NO production in fpECs. EC and EPE fpECs (n = 4 biological replicates/group) were seeded on 1% gelatin coated 6-well plates and grown under the same conditions as described above until 80-100% confluence. At the day of the experiment, fpECs were treated with 5 µM Yoda1 and/or 10 nM GSK or DMSO as a vehicle control added to the media for 3 min and 10 min, respectively. Time point were based on preliminary time course experiments which identified the strongest upregulation of p-eNOS upon treatment. Immediately afterwards protein was harvested by scraping of the cells on ice in RIPA buffer (Sigma Aldrich) with cOmplete Mini proteinase inhibitor cocktail (Roche) and Phosphatase Inhibitor Cocktail II (ThermoFisher Scientific). Protein concentration was quantified by Pierce BCA Protein Assay (ThermoFisher Scientific). Samples were denatured at 95°C in Laemmli buffer, and 40 µg were loaded in a 4-20% Mini-PROTEAN TGX Gel (Bio-Rad Laboratories, Hertfordshire, UK) for gel electrophoresis. Protein was transferred to a PVDF membrane using a Trans-Blot Turbo Transfer System (Bio-Rad Laboratories). Membranes were blocked with 5% bovine serum albumin (BSA) in tris-borat-EDTA buffer with 0.1% tween (TBE-T) and incubated overnight at 4°C with the following primary antibodies diluted in 5% BSA in TBE-T: eNOS 1:3000 (BD Biosciences; #610297; RRID:AB_397691), p-eNOS 1:500 (Cell Signaling Technology, Danvers, MA, USA; #9571; RRID:AB_329837), and GAPDH 1:5000 (NovusBio; #NB300-328; RRID:AB_10000531). Primary antibodies were detected with a secondary antibody anti-mouse diluted 1:10.000 in 5% BSA in TBE-T (Bio-Rad, #170-6516; RRID:AB_11125547) or anti-rabbit diluted 1:1000 in 2.5% milk in tris-buffered saline with 0.05% tween (Cell Signaling Technology, #7074; RRID:AB_2099233), respectively. For p-eNOS, 0.5% Phosphatase Inhibitor Cocktail II was added during blocking and antibody incubation. To re-probe the same membrane with different antibodies membranes were stripped with Restore Western Blot Stripping Buffer (ThermoFisher Scientific) for 20 min at room temperature. Visualization and imaging was done by chemiluminescence on a Fusion FX6 machine with the Evolution-Capt Software (Vilber Lourmat SAS, Marne-la-Vallée, France). Validation of the antibodies is shown in Fig. S1. Quantification was done in ImageJ. After merging of the blots and background correction the built-in ‘Analyze Gels’ function and the percentage measurements of the plotted lanes were used. All samples were run in technical duplicates. Statistical outliers were removed using the ROUT method in GraphPadPrism version 9.5.1. (GraphPad Software, Boston, USA) and duplicates were averaged for statistical analysis.

### Electric Cell-Substrate Impedance Sensing (ECIS)

ECIS was used to investigate the influence of Piezo1 and/or TRPV4 activation on the endothelial barrier integrity. Basal measurements are described in the supplementary material (Document S1). FpECs from EC (n = 3) and EPE (n = 4) donors were seeded in technical duplicates on L-cysteine pre-treated, 1% gelatin-coated 8-well, gold-plated electrode arrays (8W10E+; Applied Biophysics, Troy, USA) in supplemented endothelial cell growth medium (Promocell) with 10% FCS and grown under the same conditions as described above throughout the experiment. Cell layer impedance was measured every 48 s throughout the experiment at multiple frequencies (4000–64000Hz) using an ECIS Model Z Theta (Applied Biophysics) with ECIS software (version 1.2.186.0 PC) to calculate resistance and capacitance. At confluence (determined by a stable capacitance baseline), cells were treated with 5 µM Yoda1 and/or 10 nM GSK or DMSO as a vehicle control in supplemented endothelial cell growth medium with 10% FCS. After 30 min cells were washed and the treatment was replaced with fresh supplemented endothelial cell growth medium with 10% FCS. 30 min was determined as a suitable treatment time as it leads to a visible effect on the fpECs, but allows cells to survive. For analysis, resistance measurements at 4000 Hz were divided by the pre-treatment baseline to obtain relative values and technical duplicates were averaged.

### Cell Proliferation Assay (MTS)

The MTS assay was performed using a CellTiter 96® AQueous One Solution kit (Promega, Madison, USA) according to the manufacturer’s instructions. One day prior to the experiment 1x10^4^ fpECs per well (n = 3 biological replicates/ group in technical triplicates) were seeded in a 96-well plate and grown under the same conditions as described above. At the day of the experiment, fpECs were treated with 5 µM Yoda1 and/or 10 nM GSK or DMSO as a vehicle control added to the media for 30 min. Immediately after, fpECs were washed with fresh media and 20 µl CellTiter 96® AQueous One Solution Reagent was added per well. Cells were incubated at 37°C, 12% O_2_ and 5% CO_2_ for 2.5 hours, after which 25 μl of 10% sodium dodecyl sulfate were added per well. Absorbance at 490 nm was measured using a CLARIOstar Plus plate reader (BMG Labtech, Ortenberg, Germany).

### Cell treatment and immunofluorescence

EC and EPE fpECs (n = 3 biological replicates/group) were seeded on 1% gelatin coated 8-well glass chamber slides and grown under the same conditions and treated as described for the MTS assay. Immediately afterwards cells were washed with PBS, fixed with 4% paraformaldehyde for 20 min at room temperature, and stored in PBS at 4°C until staining. For staining, cells were permeabilized with 0.2% triton-X-100 in PBS and blocked with 5% BSA in PBS for 1 hour at room temperature. A rabbit monoclonal primary antibody anti-VE-cadherin (1.13µg/ml, Cell Signaling Technology, #D87F2; RRID:AB_10839118) was applied diluted 1:100 in PBS with 0.5% BSA for 1 hour at room temperature and moved to 4°C overnight. As secondary antibody goat anti-rabbit IgG DyLight 550 (5 µg/ml, Invitrogen, ThermoFisher Scientific, #84541; RRID:AB_10942173) was applied, diluted 1:200 in PBS with 0.5% BSA together with Alexa Fluor 488 Phalloidin (diluted 1:1000, Invitrogen, ThermoFisher Scientific) for 1 hour at room temperature. DAPI was used to counterstain the nuclei. Irrelevant rabbit IgG and omitting of the primary antibody were used as negative controls. A 3.5x3.5 mm sized area for each condition was imaged with an Olympus SLIDEVIEW VS200 slide scanner (20x objective, Olympus).

### Reactive oxygen species (ROS) measurements

Flow cytometry was performed to detect ROS levels of EC and EPE fpECs (n = 5 biological replicates/group). Cells were plated at a density of 0.5x10^6^ cells/well in 1% gelatin coated 6-well plates. After 24 hours, CellROX Green Reagent (Invitrogen) was added to each well at a concentration of 375 nM and incubated for 30 min under the same growth conditions as specified. Cells were then trypsinized and resuspended in PBS + 3% FCS. Staining was performed according to the manufacturer’s instructions. 5 µL of 7-AAD viability dye (BD Biosciences) was added to each stained tube right before flow cytometry measurement. 5x10^4^ cells per sample were counted and separated by size using FSC and SSC, followed by doublet discrimination and gating against ROS – FITC area and viability marker. An unstained sample was included for gating and the same gating strategy was employed on all investigated samples (Fig. S2). ROS and 7AAD staining were compensated by individual staining on EC fpECs. To induce a positive ROS signal, 100 µM menadione was used. To induce a positive 7AAD signal, cells were treated with 70% Ethanol for 15 min. Measurements were performed using a CytoFLEX SI flow cytometer and analyzed with FlowJo™ v10.9 software (RRID:SCR_008520).

### Statistical analysis

All data were tested for normal distribution using the Shapiro-Wilk test in order to choose the appropriate statistical tests. All used statistical tests and group sizes are specified in the respective legend of figures or tables. P-values < 0.05 were considered statistically significant. Except from the RNA-Seq data analysis (specified above), all statistical tests were conducted with GraphPadPrism (version 9.5.1.; RRID:SCR_002798).

## Results

### *PIEZO1* and *TRPV4* are co-expressed in fpECs

**Figure 1:**
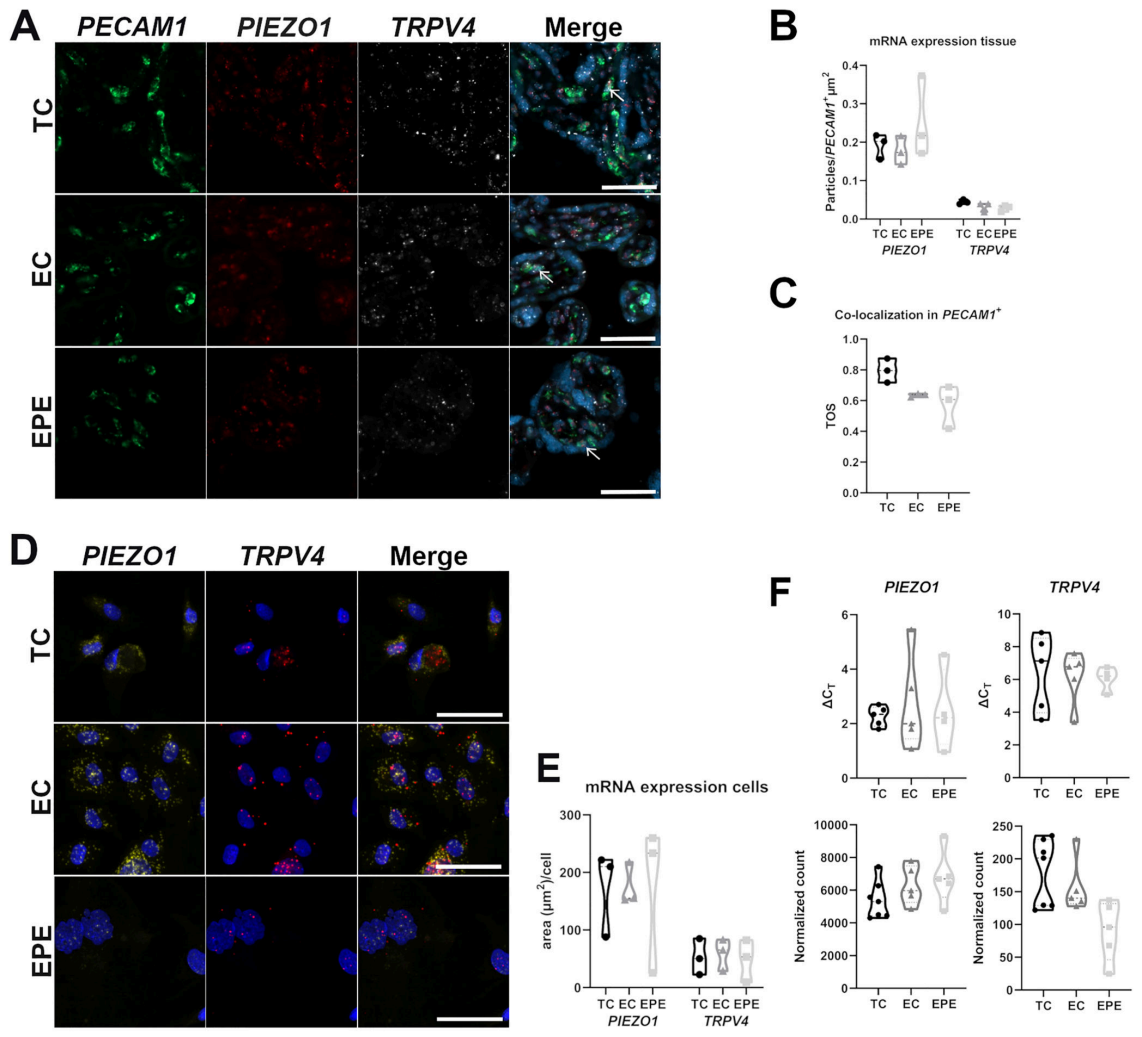
mRNA expression of *PIEZO1* and *TRPV4* in placental tissue and feto-placental endothelial cells (fpECs). **A –** Representative images of *PIEZO1* (red) and *TRPV4* (white) expression in intact placental tissue visualized by *in-situ* hybridization. *PECAM1* (green) was used as fpEC marker. Arrows exemplarily mark fpECs co-expressing *PECAM1, PIEZO1* and *TRPV4*. DAPI (blue) marks nuclei. Scale bars = 50 µm. TC = term controls, EC = early controls, EPE = early-onset preeclampsia. **B** – Violin plot showing the quantification of mRNA particles per *PECAM1*^+^ µm^2^ in placental tissue sections for each group. Kruskal-Wallis test + Dunn’s post hoc test, not significant. n = 3 biological replicates/group. **C** – Violin plot showing the threshold overlap score (TOS) of *PIEZO1* and *TRPV4* within the *PECAM1*^+^ area in placental tissue sections. Ordinary one-way ANOVA + Tukey’s post hoc test, not significant. n = 3 biological replicates/group. **D -** Representative images of *PIEZO1* (yellow) and *TRPV4* (red) expression visualized by *in-situ* hybridization in isolated fpECs. DAPI (blue) marks nuclei. Scale bars = 50 µm. See also Figure S1. **E** – Violin plot showing the quantification of mRNA as stained area (µm^2^) per cell in isolated fpECs for each group. Ordinary one-way ANOVA + Tukey’s post hoc test, not significant. n = 3 biological replicates /group. **F** – Violin plots showing the expression levels of *PIEZO1* and *TRPV4* as ΔC_T_ measured by qPCR (top, n = 4-5 biological replicates/group) and as normalized counts measured by RNA-Seq (bottom, n = 5-7 biological replicates/group. Kruskal-Wallis + Dunn’s post hoc test, not significant.

ISH showed that mRNA of both channels is expressed by *PECAM1*^+^ fpECs in placental tissue (Fig. 1A), while the expression levels did not differ between TC, EC, and EPE fpECs (Fig. 1B). Quantification of the co-localization of *PIEZO1* and *TRPV4* after ISH confirmed that both channels are frequently expressed within the same fpEC in placental tissue sections (mean TOS of all samples = 0.67±0.13), with no difference between the groups (Fig. 1C). Co-expression of mRNA of both channels was also observed and confirmed in cultured fpECs by ISH (Fig.1D and Fig. S3) with no difference between the groups (Fig. 1E). Quantification of mRNA levels in isolated fpECs by qPCR and by RNA-Seq confirmed the expression of both channels and also showed no significant differences between the three groups (Fig. 1F). Furthermore, expression levels did not correlate with maternal BMI, fetal weight percentile, placental weight, or fetal sex (data not shown).

### Cytosolic Ca^2+^ increase induced by Piezo1 or TRPV4 activation is reduced in EPE fpECs

**Figure 2:**
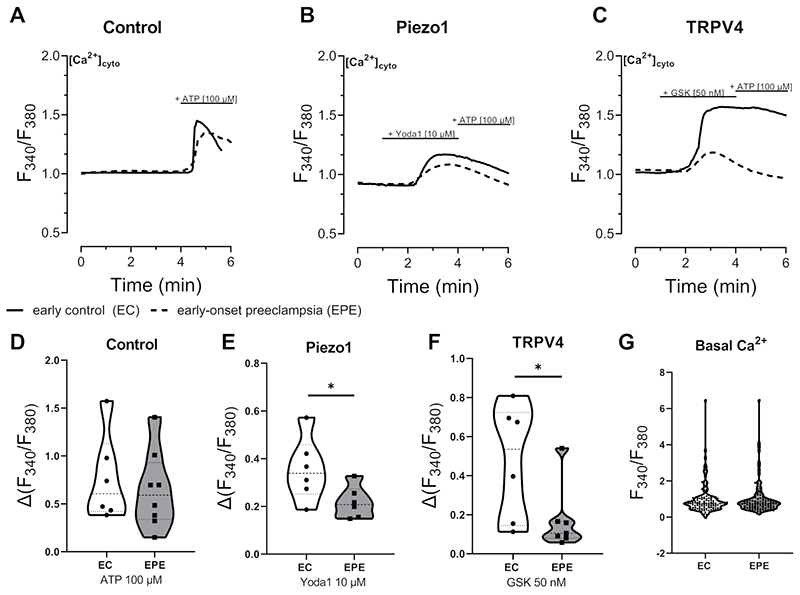
Functionality of Piezo1 and TRPV4 in early control (EC) compared to early-onset preeclamptic (EPE) feto-placental endothelial cells (fpECs). **A-C –** Mean traces of Ca^2+^ imaging experiments using EC fpECs (solid line) or EPE fpECs (dotted line). At the indicated time points, ATP (100µM) (A), Yoda1 (10µM) (B), or GSK1016790A (GSK; 50nM) (C) were added to the cells. **D - F** – Violin plots comparing the maximum amplitude from Ca^2+^-baseline measured in response to each compound between all measured EC fpECs and EPE fpECs. n = 6 measurements (3 biological replicates/group measured in technical duplicates) with a minimum of 28 cells total. Unpaired t-test, * p < 0.05; control not significant. **G** – Violin plot showing the comparison of cytosolic Ca^2+^-baseline of all measurements before addition of compounds between EC fpECs and EPE fpECs. n = 3 biological replicates/group with a minimum of 111 measurements. Unpaired t-test, not significant. Error bars = SEM.

To test whether the functionality of Piezo1 and TRPV4 are affected in EPE fpECs, we measured the cytosolic Ca^2+^ dynamics in response to chemical channel activation by either Yoda1 (Piezo1 agonist) or GSK (TRPV4 agonist) in EC compared to EPE fpECs (Fig. 2A-C). The peak response to ATP, as a positive control, was not significantly different between the groups (p=0.61; Fig. 2D). However, the rise of cytosolic Ca^2+^, induced by either Yoda1 (p=0.047; Fig. 2E) or GSK (p=0.041; Fig. 2F) was significantly reduced in EPE fpECs. To check whether this was caused by different basal Ca^2+^ levels, we compared the cytosolic Ca^2+^ levels before addition of an agonist and did not observe a difference between EC and EPE fpECs (p=0.69; Fig. 2G).

### Pre-activation of TRPV4 reduces membrane depolarization by Piezo1 activation

**Figure 3:**
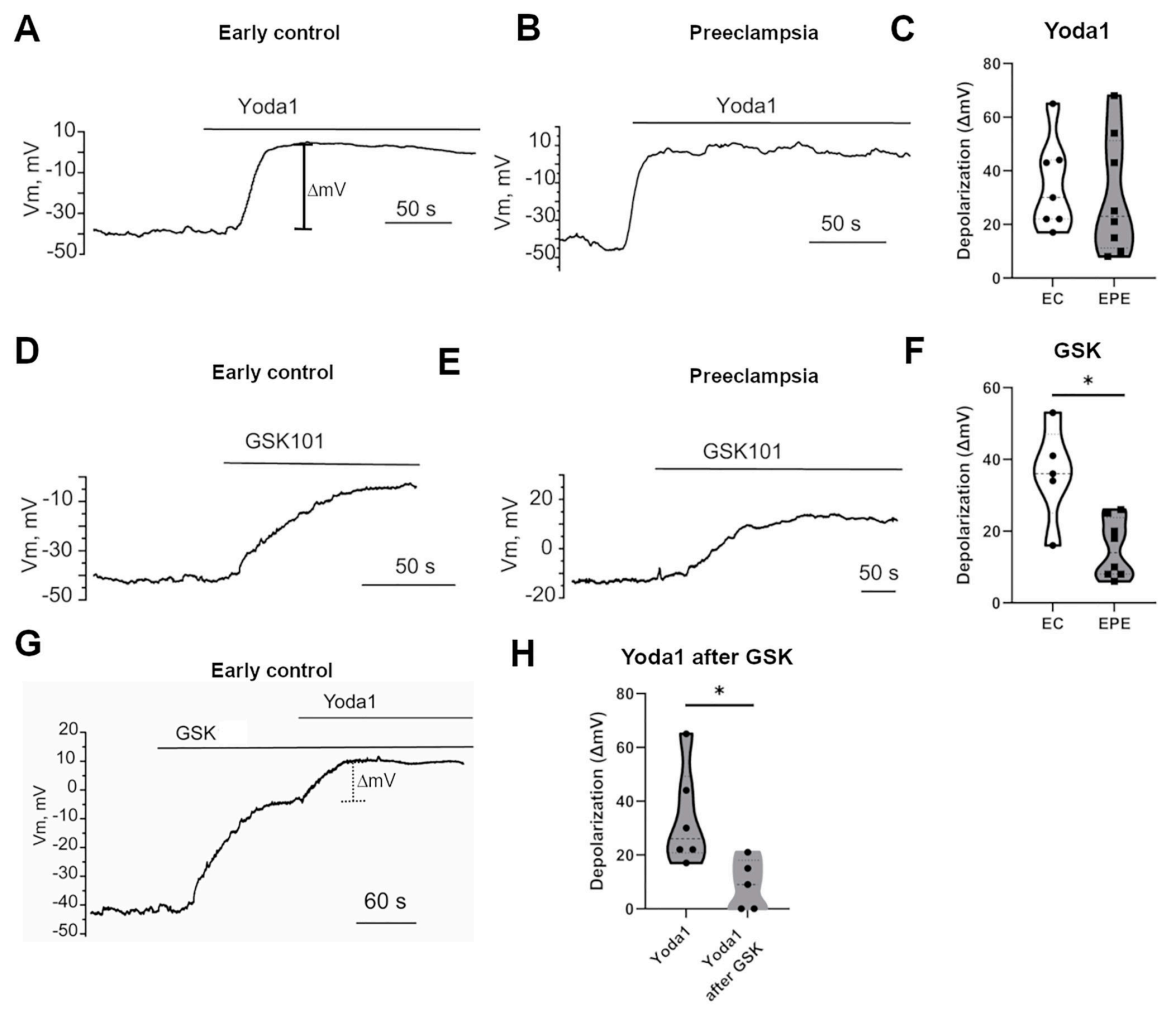
Membrane potential responses of feto-placental endothelial cells (fpECs) after activation of Piezo1 and/or TRPV4. **A, B -** Representative traces of the fpEC membrane potential responses induced by addition of 5µM Yoda1 to BAPTA-AM treated control (EC) fpECs (A) and early-onset preeclamptic (EPE) fpECs (B). The bar above indicates time of Yoda1 exposure. The bar (ΔmV) exemplarily indicates the magnitude of cell depolarization plotted in (C). **C –** Violin plot with dots indicating the magnitudes of depolarizing responses induced by addition of Yoda1 to EC fpECs and EPE fpECs in individual experiments. n = 7-8 recordings of 3-4 biological replicates/group. Welsh’s t-test, not significant. **D, E –** Equivalent Representative traces of the membrane potential responses induced by addition of 10nM of the TRPV4 agonist GSK1016790A (GSK) to EC fpECs (D) and EPE fpECs (E). **F -** Violin plot with dots indicating the magnitudes of depolarizing responses induced by addition of GSK to EC fpECs and EPE fpECs in individual experiments. n = 5-8 recordings of 3 biological replicates/group. Welsh’s t-test, * p <0.05. **G -** Representative trace of the membrane potential response of EC fpECs induced by administration of GSK (10nM) followed by the combined application of GSK (10nM) and Yoda1 (5µM). The dotted bar indicates the ΔmV plotted in (H). **H -** Violin plot comparing the maximum ΔmV in response to Yoda1 from baseline (as shown in (A)) or after previous treatment with GSK (as shown in (G)) of EC fpECs. n = 5-6 recordings of 3 biological replicates. Welsh’s t-test, * p < 0.05.

Ion fluxes across the membrane alter the membrane potential of the cell. Since Piezo1 and TRPV4 are not Ca^2+^-selective and allow the passage of other ions, we used the patch-clamp technique to investigate the extent to which channel activation affects the membrane potential of EPE and EC fpECs. Importantly, different subtypes of Ca^2+^-dependent K^+^ channels that may influence a pure depolarizing effect induced by net influx of cations are expressed by endothelial cells (19, 51), and are likely the subject of remodeling in PE. Therefore, in order to prevent their downstream activation, the cytosolic Ca^2+^ was chelated by cell pre-incubation in a BAPTA-AM containing solution, a maneuver previously used by us (50). In contrast to the Ca^2+^ imaging experiments, the mean membrane depolarization (ΔmV) induced by Yoda1 was not significantly different between the groups (EC: 34.7±6.4 ΔmV; EPE: 30.5±7.8 ΔmV; p=0.68, Fig. 3A-C). In line with the reduced Ca^2+^ flux, the depolarization induced by GSK was significantly lower in EPE fpECs (EC: 36.0±6.0 ΔmV; EPE: 15.13±2.9 ΔmV; p=0.02, Fig. 3D-F). In a next step, we used EC fpECs to test the hypothesis that a co-regulating relationship between both channels exists in fpECs and, in particular, that pre-activation of TRPV4 can reduce the Piezo1-mediated membrane depolarization. Indeed, TRPV4 activation by GSK prior to application of Yoda1 significantly decreased Yoda1-induced depolarization (Yoda1 alone: 33.3±7.4 ΔmV; Yoda1 after GSK: 9±4.1 ΔmV; p=0.02, Fig. 3G+H).

### Activation of Piezo1, but not TRPV4, induces endothelial nitric oxide synthase (eNOS) phosphorylation in fpECs

**Figure 4:**
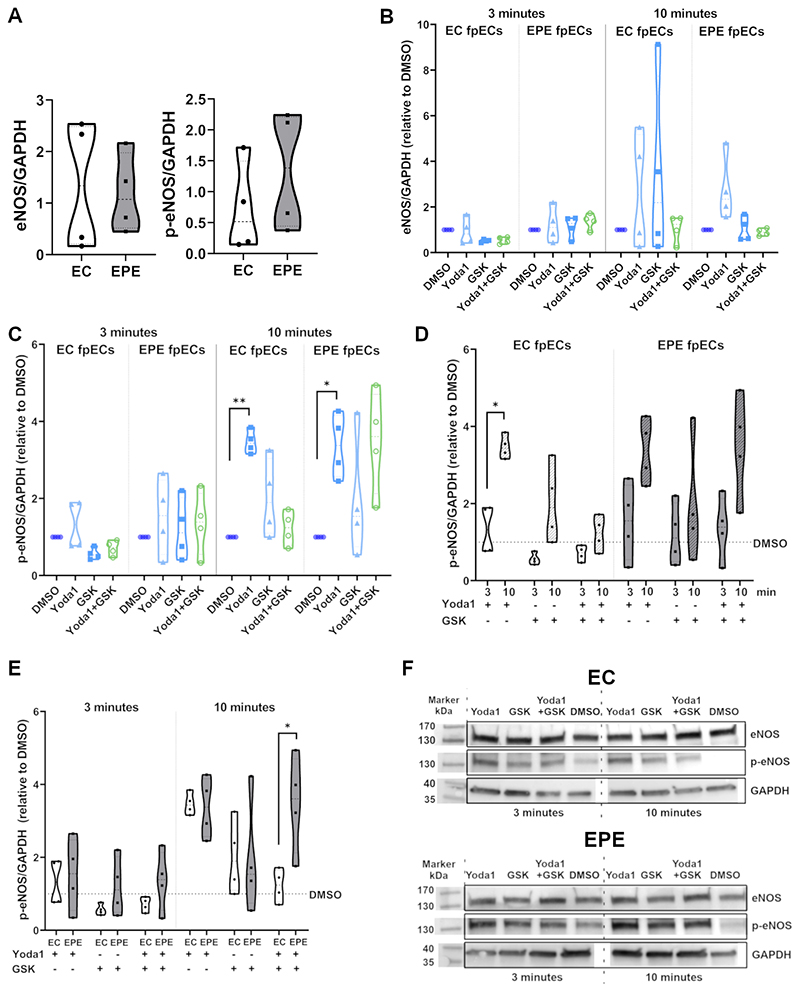
Activation of Piezo1 induces phosphorylation of endothelial nitric oxide synthase (eNOS) in feto-placental endothelial cells (fpECs). **A -** Violin plots showing the basal levels (pooled DMSO vehicle controls) of eNOS (A) and phosphorylated eNOS at Ser1177 (p-eNOS) (B) relative to the housekeeper GAPDH in early control (EC) fpECs compared to early-onset preeclamptic (EPE) fpECs. n = 4 biological replicates/group. One-Way ANOVA + Sidak’s post-hoc test, not significant. **B + C -** Violin plots showing eNOS (B) and p-eNOS (C) levels after treatment with the Piezo1 agonist Yoda1 (5µM) and/or the TRPV4 agonist GSK1016790A (GSK; 10nM) relative to DMSO controls. n = 4 biological replicates/group. Repeated-measures one way ANOVA + Dunnett’s post-hoc test, * p < 0.05, ** p < 0.01; other pairwise comparisons with DMSO not significant. **D** - Violin plot showing p-eNOS levels after treatment with Yoda1 and/or GSK after 3 minutes compared to 10 minutes of treatment. n = 4 biological replicates/group. Repeated-measures one way ANOVA + Sidak’s post-hoc test, * p<0.05; other pairwise comparisons between 3 and 10 min not significant. **E -** Violin plot showing p-eNOS levels after treatment with Yoda1 and/or GSK after 3 and 10 minutes compared between EC and EPE fpECs. n = 4 biological replicates/group. Repeated Measures One-Way ANOVA + Sidak’s post-hoc test, * p < 0.05; other pairwise comparisons between EC and EPE not significant. **F** – Representative examples of Western Blots showing bands for eNOS and p-eNOS at ~140 kDa and for GAPDH at ~37 kDa.

To investigate whether the observed differences in Ca^2+^ fluxes and membrane potential in fpECs are translated to altered functionality, we investigated the phosphorylation of eNOS at Ser1177 as a measure of cellular NO production. Since this and other methods used in the following experiments do not allow for the same temporal resolution of events like patch-clamping, cells were treated with Yoda1 and GSK simultaneously instead of consecutively. Under basal conditions, i.e. in DMSO vehicle controls, the level of total eNOS and phosphorylated eNOS (p-eNOS) did not differ between EC and EPE fpECs (Fig. 4A). Treatment for 3 or 10 min with Yoda1 and/or GSK did not induce a significant change in total eNOS levels (Fig. 4B). After a treatment period of 10 min, Yoda1 alone induced a significant increase in p-eNOS levels compared to DMSO controls in both groups (p = 0.01 for EC; p = 0.02 for EPE; Fig. 4C). At this time point, an increase of the average p-eNOS levels compared to DMSO controls was observed in both groups for GSK or combined treatment as well, but this was not significant (mean values 1.23 – 3.48; Fig. 4C). The effect of Yoda1 was time-dependent and not seen after just 3 min of treatment. In EC fpECs this was reflected by a significant increase of p-eNOS protein after 10 min compared to 3 min of treatment (p = 0.04, Fig. 4D). When comparing EC and EPE fpECs, 10 min of treatment with GSK or Yoda1 alone did not lead to significantly different p-eNOS levels between the groups. However, combined treatment with Yoda1 and GSK induced a significantly higher increase of p-eNOS levels relative to DMSO controls in EPE compared to EC fpECs (p = 0.02, Fig. 4E).

### Only combined activation of Piezo1 and TRPV4 reduces fpEC barrier integrity, with a stronger effect in EPE fpECs

**Figure 5:**
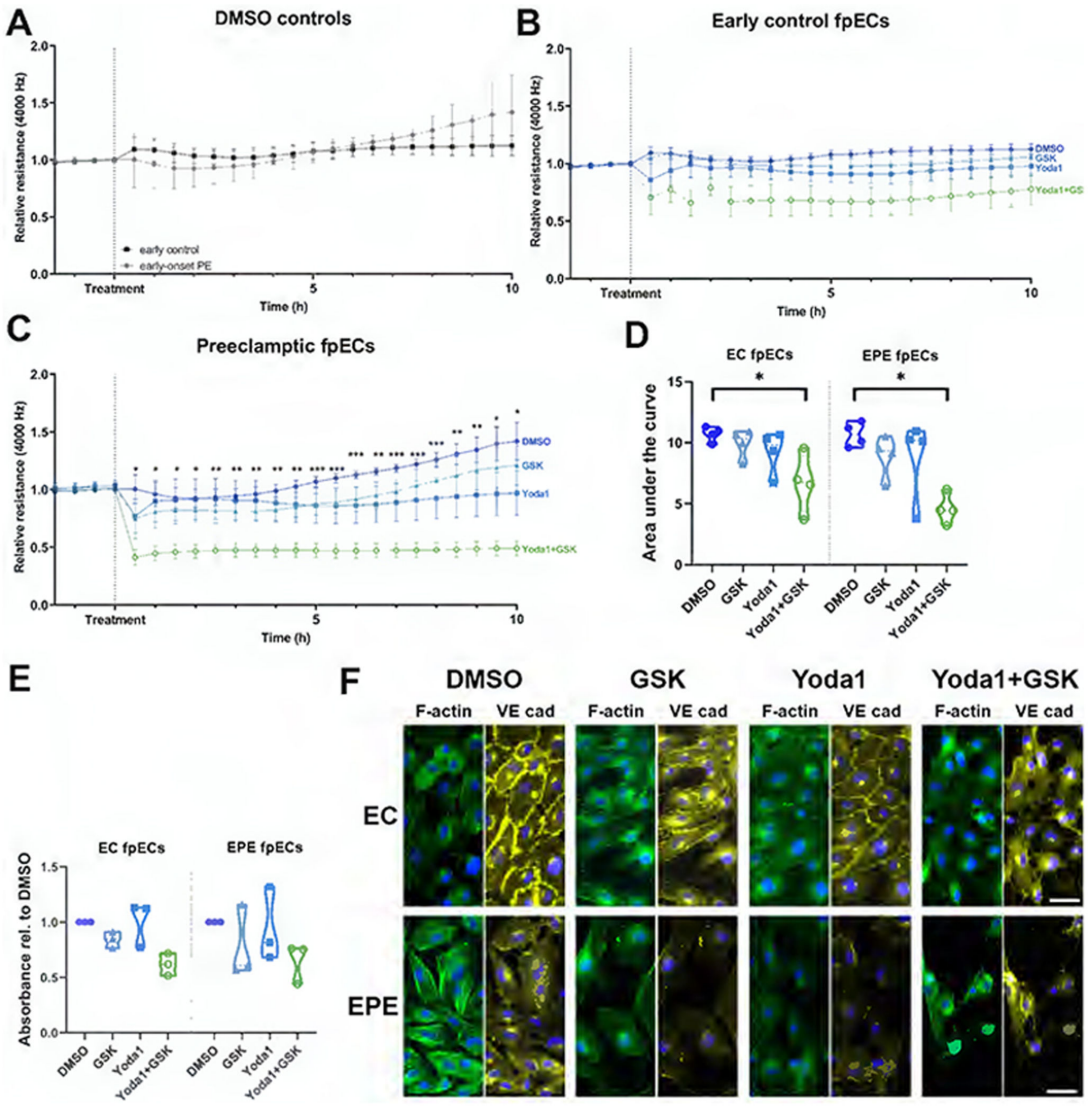
Barrier integrity of feto-placental endothelial cells (fpECs) is compromised by combined activation of Piezo1 and TRPV4. **A – C** – Graphs showing the relative endothelial barrier resistance measured over time as mean±SEM. n = 4 biological replicates/group measured in technical duplicates. **A** - DMSO controls do not differ between early controls (EC) and early-onset preeclamptic (EPE) fpECs. Repeated measures two-way ANOVA, not significant. **B+C -** Combined treatment with 5µM Yoda1 and 10nM GSK1016790A (GSK) significantly reduces the resistance from 0.5 hours post-treatment in EPE fpECs only. Repeated measures two-way ANOVA + Dunnett’s post hoc test; * p < 0.05, ** p < 0.01, *** p < 0.001 between DMSO and Yoda1 + GSK. **D** – Violin plot showing the comparison of the area under the curve over 10 hours after the treatment shown in (B) and (C). Kruskal-Wallis test + Dunn’s post hoc test; * p < 0.05; other pairwise comparisons with DMSO not significant. See also Figure S3. **E** – Violin plot showing the results of the MTS cell proliferation assay as absorbance measured at 490 nm relative to the DMSO control. n = 3 biological replicates/group measured in technical triplicates. Friedman test, not significant. **F** - Representative examples of DMSO controls and fpECs treated 30 min with 5µM Yoda1 and/or 10nM GSK. FpECs are stained for F-actin (phalloidin, green), VE-cadherin (VE cad, yellow), and nuclei (DAPI, blue). Scale bars = 50 µm. See also Figure S4.

As a further important measure of endothelial functionality, we investigated the influence of channel activation on endothelial barrier integrity using ECIS. Therefore, EC and EPE fpECs were treated with Yoda1 and/or GSK and DMSO as vehicle control, while monitoring the resistance of the cell monolayer. The relative resistance of the DMSO controls did not differ between the groups (Fig. 5A). In both groups, only the combined treatment with Yoda1 and GSK significantly reduced the barrier integrity across 10 hours after treatment compared to the vehicle control (EC p=0.014; EPE p=0.016; Fig. 5B-D). However, only in EPE fpECs the difference to vehicle controls was significant at any time point from 0.5 hours after treatment (Fig. 5C), suggesting a more pronounced effect of combined Piezo1 and TRPV4 activation on EPE fpECs than on EC fpECs.

In general, the integrity of the endothelial cell barrier might be influenced by several factors, e.g., cell morphology and coverage. To assess whether the observed differences in placental barrier were influenced by potentially reduced cell proliferation, we performed an MTS proliferation assay upon treatment with the respective channel agonists. In line with the ECIS results, we observed a similar pattern of the agonists reducing proliferation rates, with the combined treatment having the largest effect, but not reaching significance in either group (Fig. 5E). To further assess whether directly barrier-mediating mechanisms are also affected by the channel activation, we examined cell morphology and expression of the adherens junction protein VE-cadherin and of F-actin fibers in EC and EPE fpECs 30 min after treatment with Yoda1 and/or GSK by immunofluorescence. After combined treatment, gaps appeared between the previously confluent cells and fpECs formed protrusions to maintain contact. This was accompanied by the appearance of more stress fibers and less pronounced VE-cadherin expression at the cell borders. Although the extent of this response to the treatment showed some variability within the groups, the EPE fpECs tended to show a more pronounced response than EC fpECs. In the EPE group, many cells detached and were lost, while the remaining cells showed a highly constricted morphology. Yoda1 treatment alone did not produce such effects in either group. In contrast, GSK treatment alone evoked effects similar to those described for the combined treatment, but they were less pronounced (Fig. 5F and Fig. S4).

Since reduced endothelial barrier integrity is described as a hallmark of PE, we also compared the basic characteristics of fpECs between the groups (Document S1). No significant differences were found between EC and EPE fpECs regarding the relative resistance over 30 hours after cell seeding, the time to reach confluence (EC 29.3h (17.1) vs. EPE 21.6h (30.1); median (range)), as well as the modelled barrier function (Rb; EC 5.8 ohm-cm^2^ (2.9) vs. EPE 4.1 ohm-cm^2^ (4.0)) and capacitance of the cell membrane (Cm; EC 1.26 microfarads/cm^2^ (0.05) vs. EPE 1.29 microfarads/cm^2^ (0.09)) at confluence. Similarly, the relative resistance across 18 hours post wounding was not significantly different (Fig. S5). Of note, the EPE group generally showed higher variation between the biological replicates.

### Modifications in AA metabolism as a possible factor underlying the Piezo1 and TRPV4-mediated fpEC dysfunction in EPE

**Figure 6:**
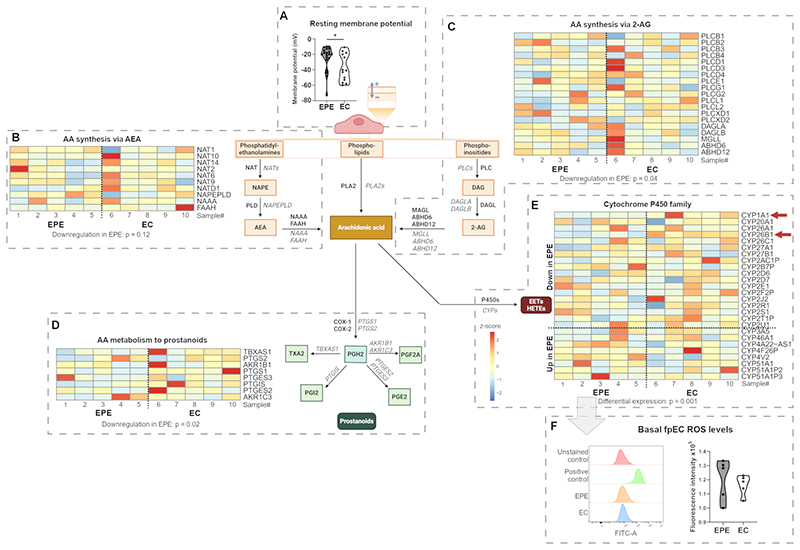
Comparison of the resting membrane potential and arachidonic acid (AA) related pathways in early-onset preeclamptic (EPE) and early control (EC) feto-placental endothelial cells (fpECs). **A -** Violin plot showing the resting membrane potential of EPE and EC fpECs recorded in whole-cell patch clamp configuration after BAPTA-AM (20µM) was added for Ca^2+^ chelation. n = 13-20 recordings of 3-4 biological replicates/group. Welsh’s t-test, * p < 0.05. **B - E –** Heat maps showing the normalized gene expression of genes encoding enzymes for (B) AA synthesis via anandamide (AEA), (C) AA synthesis via 2-arachidonoylglycerol (2-AG), (D) AA downstream metabolism to prostanoids, and (E) the cytochrome P450 family (P450s). Arrows mark overall differentially expressed P450s. All data shown in heat maps was generated by bulk RNA-Seq analysis. Each sample number (#) represents one biological replicate. P-values relate to the respective fry gene set test. See also Figure S5. **F –** Representative flow cytometry histogram and summarized violin plot showing the basal levels of reactive oxygen species (ROS) in fpECs. n = 5 biological replicates/group. Welsh’s t-test, not significant. See also Figure S5. NAT = N-acyltransferase, NAPE = N-acylphosphatidylethanolamine, PL = phospholipase, NAAA = N-acylethanolamine acid amidase, FAAH = fatty acid amide hydrolase, DAG = diacylglycerol, DAGL = diacylglycerol lipase, MAGL = monoacylglycerol lipase, ABHD = α/β-hydrolase domain containing, COX = cyclooxygenase, PG = prostaglandin, TXA2 = thromboxane, EETs = epoxyeicosatrienoic acids, HETEs = hydroxyeicosatetraenoic acids.

Endothelial dysfunction under many pathologies, including hypertension (52), atherosclerosis (19), and diabetes (53), is frequently associated with lower membrane potential values of unstimulated endothelial cells. We found here, that under the condition of intracellular Ca^2+^ buffering, unstimulated EPE fpECs had a significantly lower mean membrane potential value (-24.4±3.5 mV) compared to EC fpECs (-37.7±4.9 mV; p = 0.04; Fig. 6A), an observation that corroborates with prior observations. By RNA-Seq, we explored the expression levels of ion channels and transporters that could drive the difference in the resting membrane potential. This comparison did not show apparent differences in expression levels (Fig. S6). Therefore, we aimed to identify the involvement of functional pathways linked to EPE and ion channel regulation.

AA is widely regarded as a pro-inflammatory signaling molecule with implications in PE (54). Of note, AA as well as some of its downstream metabolites can directly interact with a variety of ion channels, including TRPV4 (36, 55). Therefore, altered AA metabolism could affect both, the resting membrane potential and other ion channel-mediated cell functions. The use of RNA-Seq enabled the comparison of gene expression in the three main pathways of AA synthesis (Fig. 6B-E). These include: i) the direct synthesis from plasma membrane phospholipids by phospholipase A2 (PLA2) (56, 57), ii) the synthesis from phosphatidylethanolamines via anandamide (AEA; Fig. 6B) (58), and iii) the synthesis from phosphoinositides via 2-arachidonoylglycerol (2-AG; Fig. 6C) (58, 59). The expression of PLA2 encoding genes (fry gene set test, downregulation p = 0.14, mixed p = 0.19; data not shown) and of genes encoding enzymes for AA synthesis via AEA did not differ between EC and EPE fpECs (fry gene set test, downregulation p = 0.12, mixed p = 0.30; Fig. 6B). In contrast, genes encoding enzymes for AA synthesis via 2-AG were significantly downregulated in EPE compared to EC fpECs (fry gene set test, downregulation p = 0.04; Fig. 6C). Additionally, genes involved in metabolizing AA into its downstream products in the placenta via cyclooxygenases (COX) (60) were also downregulated in EPE fpECs (fry gene set test, downregulation p = 0.02; Fig. 6D). Furthermore, the expression of genes belonging to the cytochrome P450 family (P450s) was observed to be significantly altered in EPE fpECs (fry gene set test, mixed p = 0.001; Fig. 6E). These genes are involved in the production of epoxyeicosatrienoic acids (EETs) and hydroxyeicosatetraenoic acid (HETEs), and can promote endothelial reactive oxygen species (ROS) production downstream of AA (61). Of note, the two genes *CYP1A1* and *CYP26B1* were included in the list of overall differentially expressed genes and were both significantly downregulated in EPE compared to EC fpECs. This dysregulation of the P450s suggests an impairment of fpEC ROS production in EPE. However, ROS levels under basal conditions were not statistically different between EC and EPE fpECs (Fig. 6F).

## Discussion

Appropriate placental vascular function ensures a constant low resistance exchange between the fetal and maternal circulation (62). To enable this, placental endothelial cells must respond to mechanical stimuli, such as shear stress, to initiate necessary adaptations of cell function and vascular tone. Here, we investigated for the first time the functionality and interaction of the mechanosensitive ion channels Piezo1 and TRPV4 and their possible functional implications in the placental endothelium affected by EPE.

Our combined *PIEZO1* and *TRPV4* expression data, together with the Ca^2+^ imaging data, demonstrate that both channels are expressed in fpECs and that their functionality is preserved when these cells are cultured *in vitro*. Piezo1 has previously been shown to mediate shear stress responses in fpECs, but the cell populations used in previous studies were predominately derived from the placental microcirculation (24). Here we demonstrate co-expression of Piezo1 and TRPV4 in the macrovasculature of the human placenta. Importantly, the patch-clamp experiments provide further evidence that both channels are active within the identical cell, thereby opening up the possibility of a combined regulatory mechanism between the channels. This is in line with several studies that have proposed a similar connection in other cells and tissues, including HUVECs, pancreatic acinar cells, chondrocytes, central nervous system capillaries, and the hepatic portal vein (27–31). Interestingly, the identified interactions of the two channels appear to differ between most of these studies, suggesting a highly specific adaptation to the microenvironment in the respective tissue. While some studies report that Piezo1 acts upstream of TRPV4 (28, 29), others do not find evidence for this (27) or show a regulatory effect depending on which channel is activated first in the experimental setup (31). However, all of the studies suggest a reciprocal functional relationship in which the activation of one channel affects the activity of the other one. Ultimately, this combined activity of both channels can fine-tune Ca^2+^-mediated downstream pathways. The decreased depolarization in response to Piezo1 activation, after TRPV4 activation, observed in the control fpECs, is consistent with what has been reported for chondrocytes (31).

With regard to the setting of EPE, the cytosolic Ca^2+^ increase in response to Piezo1 or TRPV4 agonists was found to be diminished in EPE fpECs. This implicates that albeit similar expression levels, the functionality of both channels is impaired. Comparable findings were reported by Steinecker-Frohnwieser et al. To the best of our knowledge, this is the only other study to investigate both channels in primary human cells derived from pathophysiological conditions, i.e., osteoarthritis. The pathological cells, like EPE fpECs, show a reduced increase in cytoplasmic Ca^2+^ through TRPV4. Furthermore, a range of downstream signaling pathways triggered by both, Piezo1 and TRPV4 agonists, are activated to a varying extent in pathological chondrocytes compared to healthy controls (31). In our study, regulation of eNOS activity was chosen as an important downstream pathway of fpEC function since both channels have been shown to induce endothelial NO synthesis (26, 63). Specifically, in the placental endothelium, activation of Piezo1 can induce phosphorylation of eNOS (25), while activation of TRPV4 has been shown to induce NO production and endothelium-dependent and –independent vasodilation (32). Partially in line with this we observed a significant time-dependent upregulation of p-eNOS in response to Piezo1 activation in EC and EPE fpECs, but no significant effect upon TRPV4 activation alone. This suggests that TRPV4 does not play a significant role in NO production by fpECs. When comparing basal control with EPE conditions, both total eNOS and p-eNOS levels did not differ between the fpEC groups. This complements previous studies that have reported either unchanged or decreased levels of eNOS protein in placental tissue or HUVECs from pregnancies affected by PE (34, 64–66). Furthermore, the increase in p-eNOS resulting from Piezo1 activation was comparable between EC and EPE fpECs, implying that the diminished Ca^2+^ flux does not impede NO production. Interestingly, the combined activation of Piezo1 and TRPV4 resulted in a significantly higher p-eNOS upregulation in EPE compared to EC fpECs. This finding was unexpected, given that the reduced response to the channel agonists in EPE, as observed by Ca^2+^ imaging and, in the case of TRPV4 also patch clamp, implied reduced rather than increased eNOS phosphorylation. Such differences were not observed when the channels were activated separately. It can thus be postulated that Piezo1-TRPV4 coupling plays a distinctive role in the EPE setting, where it may be subject to alterations and enhanced downstream signaling, resulting in elevated NO production. A potential explanation for this is a compensatory mechanism. It is well documented that NO bioavailability is reduced in PE, mainly induced by oxidative stress and subsequent reduced substrate availability and post-translational modifications (67). Consequently, Piezo1 and TRPV4 may co-regulate higher NO production to counteract this effect in EPE fpECs.

Since reduced barrier integrity is a hallmark of endothelial dysfunction and implicated in PE (8), the influence on endothelial barrier integrity was chosen as an important functional parameter in this study. Under basal conditions, a reduction in barrier integrity was not evident for EPE fpECs. However, this may be attributed to the fact that cells from both groups were cultivated under identical standard conditions, which do not reflect the *in vivo* PE (micro)environment (i.e., increased inflammatory cytokines, hypoxia (68, 69)). Interestingly, only when both channels were activated by agonists simultaneously we observed a significantly reduced barrier integrity, independent of the fpEC phenotype. While this was at least partially mediated by the reduction of VE-cadherin at cell-to-cell junctions, the combined treatment also resulted in a reduction in the proliferation rate of fpECs. Albeit not significant, this is in line with a previous study reporting reduced proliferation only by combined channel activity in osteoblasts (70). This further supports the hypothesis of a co-regulating relationship between both channels and implies functionally relevant consequences of this interdependence. Interestingly, GSK alone had a similar effect on cell morphology and VE-cadherin expression of fpECs, which did not seem to be reflected in the barrier integrity measurements. In contrast to our data, it has been shown in HUVECs that treatment with Yoda1 alone is sufficient to reduce VE-cadherin adherens junctions and to induce F-actin disruption (28). Although this earlier study did not investigate combined treatment, Swain et al. observed that blocking TRPV4 prevented both the VE-cadherin loss and F-actin disruption induced by Piezo1 activation, further supporting the idea of both channels co-regulating barrier integrity. Additionally, in a different study on pancreatic acinar cells, the same group showed that in these cells, only activation of both channels is sufficient to induce pathological events (29), which is in line with what we observed in fpECs.

The mechanism linking Piezo1 to VE-cadherin and F-actin in ECs has recently been proposed to consist of two pools of Piezo1 in the cell, an apical and a junctional pool. At the cell-to-cell junction, Piezo1 can interact directly with VE-cadherin (71). This suggests that direct interactions of Piezo1 with the adherens junctions and focal adhesions/F-actin might affect endothelial barrier integrity in the placenta. The coordination and fine-tuning between those Piezo1 pools has been proposed to occur via regulation of the local Ca^2+^ levels (71). This mechanism might explain the stronger impairment of the endothelial barrier in response to combined channel activation when opening of TRPV4 would lead to additional Ca^2+^ influx. Importantly, the effect of combined channel activation on barrier integrity was more pronounced in EPE fpECs. As with eNOS phosphorylation, this finding seems to contradict the reduced increase in intracellular Ca^2+^ levels through both channels observed in EPE fpECs. This discrepancy could be attributed to the specific characteristics of EPE fpECs and their susceptibility to such Ca^2+^-mediated barrier disruption, and it also reinforces the idea of altered mechanisms coupling Piezo1 and TRPV4 in EPE. At the level of membrane depolarization, the reduction in channel functionality was only significant for TRPV4. Together with the morphological changes observed upon GSK treatment, this suggests a potentially more important mechanistic role for TRPV4 mediating barrier integrity in the EPE setting than Piezo1; however, activation of *both* channels was required to induce the reduction in barrier integrity in both groups.

Apart from the differences in Ca^2+^ flux, our patch-clamp experiments revealed that under conditions of intracellular Ca^2+^ buffering, EPE fpECs have a lower resting membrane potential than EC fpECs. This implies differences in cell membrane structure or functionality. We, therefore, further aimed to identify possible mechanisms that could be causative for both the altered functionality of Piezo1 and TRPV4 and the lower resting membrane potential in EPE fpECs. Due to the buffering, Ca^2+^-activated channels, such as the Ca^2+^-activated potassium channels, which are involved in the control of membrane potential in most cell types (72), do not appear to be responsible for the observed difference. We also found no significant difference in the expression levels of other ion channels by RNA-Seq. However, this does not exclude the possibility of altered channel /transporter functionality that control the membrane potential, including Na^+^,K^+^ ATPAse (73), Na^+^/Ca^2+^ exchanger (50), inwardly rectifying K^+^ channels and nonselective Ca^2+^ channels (74) not investigated in the current study. Involvement of such mechanisms has previously been suggested to be the case in HUVECs from PE pregnancies, which show altered membrane permeability to several multivalent cations (Ca^2+^, Mn^2+^, Ba^2+^) compared to controls. In contrast to our observations in fpECs, this study on HUVECs also reports elevated basal Ca^2+^ levels and decreased histamine-stimulated Ca^2+^ influx in PE. Differences between the umbilical and placental endothelial bed (75) and variability within the entity of PE cases (76) are plausible explanations for this functional difference. Therefore, we used the generated RNA-Seq dataset to identify possible pathways that could regulate ion channel functionality and, thereby, the resting membrane potential. We focused on key enzymes of AA metabolism as it is involved in both, ion channel activation and in the pathophysiology of PE (36, 55). The significant downregulation of genes encoding enzymes that induce the sequential hydrolysis of AA-containing phosphoinositides via 2-AG suggests a reduced bioavailability of AA in EPE fpECs. Additionally, Piezo1 has previously been shown to activate PLA2 (29, 77). Therefore, the reduced Piezo1-mediated Ca^2+^ influx observed in the EPE fpECs may lead to reduced activity of PLA2, further contributing to reduced AA bioavailability.

Downstream of AA, Piezo1 and TRPV4 have been shown to induce the production of several prostanoids, such as prostaglandin E2 (PGE2), D2 (PGD2), and F2α (PGF2A) (32, 77, 78). Thus, the downregulation of the genes that metabolize AA to prostanoids in EPE fpECs is consistent with the reduced channel functionality and suggests an overall reduced conversion of AA to prostanoids. Importantly, it has been implicated that differences in AA tissue levels lead to preferential activation of different AA downstream pathways, with low AA levels enhancing the production of pro-inflammatory eicosanoids such as PGE2 (54). Besides prostanoids, EETs and HETEs catalyzed by the P450s are also derived from AA. The differential expression of genes encoding P450s, with some up regulated and some down regulated in EPE fpECs, suggests dysbalances within this AA downstream pathway. HETEs and EETs affect a variety of ion channels (61), including 5’,6’-EET as a direct activator of TRPV4 (36). Therefore, dysbalanced P450s might contribute to altered ion channel functionality in EPE fpECs. Furthermore, our results point in the same direction as a previous study on human umbilical artery smooth muscle cells from PE pregnancies. These cells show an increased Ca^2+^ influx in response to AA as a consequence of an increased activity of the P450 pathway and decreased activity of the COX and lipoxygenase pathways compared to healthy controls (79). Among the P450s, *CYP1A1* and *CYP26B1*, were in the list of overall significantly differentially expressed genes, and both were downregulated in EPE compared to EC fpECs. Interestingly, a combined knockdown of *CYP1A1* and *CYP26B1* results in reduced angiogenesis in HUVECs (80), implying a possible role in the impairment of placental vascular structure and function in PE. Furthermore, *CYP1A1* encodes an AA hydroxylase with 19-HETE as the main product (81). 19-HETE has been shown to act as a vasodilator (82), hence, downregulation of *CYP1A1* may also contribute to the vasoconstrictive phenotype in EPE.

Another important aspect of AA metabolism is that reactions catalyzed by the P450s lead to the production of ROS and autoxidation of AA (61). This induces the production of lipid hydroperoxides, which can perturb both, the cell membrane structure and ion channel functionality, thereby altering cell membrane potential (61, 83, 84). The observed lower resting membrane potential together with the dysbalances in the P450s in EPE fpECs, therefore, implies an effect on the regulation of oxidative cell stress previously described in PE compromised placental tissue (85). Interestingly, we did not observe increased ROS levels in the EPE fpECs under basal conditions. Basal conditions were chosen in order to avoid an additional induction of the oxidative stress response. Thus, these results do not exclude the possibility that EPE fpECs have to cope with a higher load of oxidative stress triggers in the *in vivo* microenvironment or have a reduced antioxidative capacity, as previously suggested in PE (86). However, untangling the effects of ROS-inducing triggers and antioxidative responses in EPE fpECs is beyond the scope of the current study. Taken together, Piezo1 and TRPV4 functionality is reduced and accompanied by impaired expression of AA metabolizing enzymes in EPE fpECs. This could potentially lead to reduced bioavailability and altered conversion of AA, ultimately affecting endothelial function. Further functional investigations of the AA metabolism and the antioxidative capacity of fpECs may help identifying the mechanisms underlying the co-regulating relationship between Piezo1 and TRPV4 in the placental endothelium.

An important strength of our study is as opposed to HUVECs, the use of primary fpECs which directly reflect the placental endothelium since the heterogeneity of the umbilical-placental vascular bed is well described (75). Furthermore, we used a cohort that allowed accurate clinical characterization of EPE as well as the use of age-matched controls and samples from both sexes. However, we acknowledge that our study has some limitations. First, the *in vitro* model we used allowed us to only investigate the endothelium and not vascular functions that include the vascular smooth muscle layer. Second, the EPE group, in particular, showed a relatively high donor-specific variability despite the standardized clinical characterization. Third, we are aware that our RNA-Seq analysis does not necessarily reflect differences in the protein or enzyme activity level but rather provides a starting point for generating new hypothesis and further investigations beyond the scope of the current study.

## Conclusion and outlook

This study demonstrates the co-expression and functionality of Piezo1 and TRPV4 within one single fpEC and their combined involvement in mediating placental endothelial NO production and barrier integrity. Furthermore, our results imply a co-regulating relationship of both channels in the placental endothelium with a functional impairment in EPE. We further demonstrate that only combined channel activation is able to induce a pathological event, i.e., a significant reduction of barrier integrity, and that EPE fpECs appear to be more vulnerable to this insult. Lastly, our RNA-Seq data suggest that a dysregulated endothelial AA metabolism might be involved in mediating the observed impairments, and the activity of respective enzymes should be investigated further in the future. In this context, it should also be considered that the lipid and, specifically, fatty acid composition of the cell membrane affects Piezo1 functionality (87). Therefore, characterization of the fpEC membrane composition in EC and EPE fpECs will help to identify further mechanisms causative to the observed differences in follow-up studies. Therapeutic targeting of both Piezo1 and TRPV4 is currently suggested in the context of various pathological conditions (88–90). In the setting of EPE, direct targeting of the channels or an attempt to balance the placental endothelial AA metabolism may be feasible scenarios. Although this remains to be seen, there is a possibility that the latter could result in the restoration of Piezo1 and TRPV4 functionality and co-regulation. Direct activation of Piezo1 in the placental endothelium could increase NO production and thereby improve placental blood flow in EPE, as already suggested in the context of small for gestational age babies (25). Therefore, a better understanding of the connection between ion channel functionality and placental vascular (patho)physiology might provide new opportunities to target and improve vascular function in EPE in the future.

## Supplementary Material

Supplementary Materials

## Figures and Tables

**Table 1:** Characteristics of the study cohort

Offspring data							
Group	Gestational days	Birth weight percentile	Placental weight (g)	Fetal sex			
**Term controls (n = 13)**	277 (22)*,^#^	64 (69)^#^	680 (300)^#^	4 f 9 m			
**Early controls (n = 10)**	252.5 (47)*	60 (94)^†^	485 (420)	5 f 5 m			
**Early-onset preeclampsia (n = 7)**	224.6 (49)^#^	19 (37)^#,†^	360 (200)^#^	4 f 3 m			
**P-values**	* 0.004 ^#^ <0.0001	0.002 ^†^ 0.03	^#^<0.0001	-			
**Maternal data**							
**Group**	**Age**	**BMI before** **pregnancy**	**sFlt-1/PlGF** **ratio**	**Systolic BP** **(mmHg)**	**Diastolic BP** **(mmHg)**	**Uric acid** **(mg/dl)**	**CRP**
**Term controls (n = 13)**	34 (13)	20.9 (7.5)	-	123 (48)^#^	74.5 (46)	-	4.0 (6.8)
**Early controls (n = 10)**	30 (17)	22.1 (6.1)	-	120 (38)^†^	78 (21)	-	4.15 (12.0)
**Early-onset preeclampsia (n = 7)**	34 (18)	19.9 (7.0)	374.3 (1300)	160 (50)^#,†^	98 (48)	7.1 (3.7)	7.2 (38.9)
**P-values**	-	-	-	^#^ 0.002 ^†^ 0.0009	-	-	-

Data presented as median (range).

* (term control (TC) vs early control (EC)),

# (TC vs early-onset preeclampsia (EPE)), and

† (EC vs EPE) denote significant differences between the groups by Kruskal-Wallis test + Dunn’s post hoc test. f = female, m = male, BMI = body mass index, sFlt-1 = soluble fms-like tyrosine kinase-1, PlGF = placental growth factor, BP = blood pressure, CRP = C-reactive protein.

## Data Availability

RNA-Seq raw data is available at https://www.ncbi.nlm.nih.gov/geo/ (GEO accession #GSE274555). Other source data for this study are not publicly available due to ethical restrictions. The source data are available to verified researchers upon request by contacting the corresponding author.

## New & Noteworthy

This study shows Piezo1 and TRPV4 co-expression and functionality within primary human feto-placental endothelial cells (fpECs), mediating nitric oxide (NO) production and barrier integrity. In early-onset preeclampsia (EPE), fpEC channel functionality and co-regulation is impaired, affecting Ca^2+^ signaling and endothelial barrier function. Combined channel activation significantly reduces endothelial barrier integrity and increases NO production in EPE. Changes in arachidonic acid metabolism are suggested as a key underlying factor mediating impaired channel functionality in EPE fpECs.
