# Supplementary material for: In vitro Examination of Piezo1-TRPV4 Dynamics: Implications for Placental Endothelial Function in Normal and Preeclamptic Pregnancies
Source: Am J Physiol Cell Physiol. Author manuscript; Available in PMC 2025 Jul 28. (PMC7617947; doi:10.1152/ajpcell.00794.2024)
Supplement: Supplementary Materials [file EMS206260-suppement-Supplementary_Materials.docx]

# SUPPLEMENTAL MATERIAL

Document S1. Figures S1 – S6and methods related to Figure S5 available at doi: 10.6084/m9.figshare.27266853 (https://figshare.com/s/1f23aa7448618b742c85).
